# Transient Stress Lymphocytosis in a Child: A Case Report and Systematic Review of the Literature

**DOI:** 10.3390/hematolrep16030042

**Published:** 2024-07-03

**Authors:** Alexander Placek, Randall Y. Chan, Maria Vergara-Lluri, Russell K. Brynes

**Affiliations:** 1 Hematopathology Section, Department of Pathology, University of Southern California Keck School of Medicine, Los Angeles, CA 90033, USA; aplacek@hpllab.com (A.P.); maria.vergara-lluri@med.usc.edu (M.V.-L.); 2 Hematology-Oncology Division, Department of Pediatrics, University of Southern California Keck School of Medicine, Los Angeles, CA 90033, USA

**Keywords:** transient stress lymphocytosis, childhood, systematic review

## Abstract

Transient stress lymphocytosis (TSL) is an under-recognized phenomenon associated with an acute stressful event such as physical trauma or various emergency medical conditions. Lymphocytosis generally resolves within several hours to days of the stressor. While most reports of TSL predominantly involve adult patients, it has only rarely been reported in pediatric patients. Here, we describe the clinical course of a 9-year-old male who developed TSL following a traumatic fall from a second-story balcony and provide a systematic literature review of TSL.

## 1. Introduction

Lymphocytosis is a common finding in hospitalized patients. In children, the causes of lymphocytosis include infection, medications, thyrotoxicosis, and lymphoproliferative disorders [1,2,3]. Rarely, lymphocytosis may be associated with inheritable causes, such as the primary immunodeficiency known as B-cell expansion with NF-κB and T-cell anergy (i.e., BENTA) due to *CARD11* mutations [3]. Distinguishing non-malignant lymphocytosis from a clonal lymphoproliferative disorder is essential and requires assessment of the clinical presentation, laboratory findings, and morphological review.

TSL, a poorly studied cause of lymphocytosis, is associated in adults with significant physical trauma or various emergency medical conditions including cardiac emergencies, gynecologic and obstetric emergencies, and sickle cell disease in crisis [4,5,6,7,8,9,10,11,12]. Resolution of the lymphocytosis typically occurs within 24–48 h and may be followed by neutrophilia. We report the case of a 9-year-old male with TSL due to serious physical trauma.

## 2. Case

**Figure 1:**
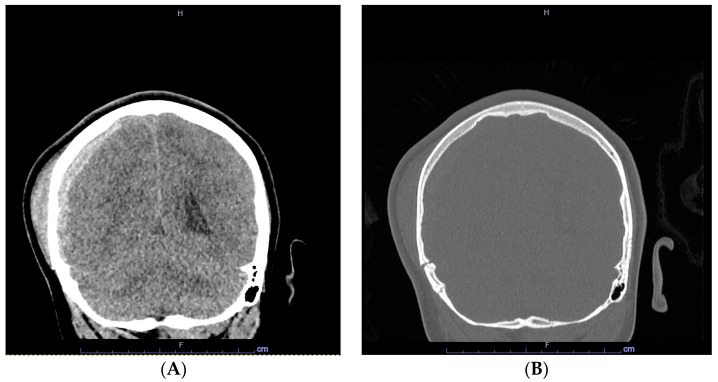
Head CT images of right acute subdural hematoma (**A**), and bone window of skull fractures (**B**).

A previously healthy 9-year-old male presented with multiple traumatic injuries after falling from a second-story balcony. On initial evaluation in the emergency department, he was tachycardic and hypertensive, with a Glasgow coma scale score of six. Computed tomography (CT) of the head revealed a right subdural hematoma measuring 9 mm with subfalcine herniation, impending transtentorial herniation, and displaced skull fractures through the occipital, bilateral parietal, and right temporal bones and superior sagittal and transverse sinus (Figure 1). CT of the thorax detected bilateral lung consolidations suggestive of aspiration, as well as a left clavicular fracture.

**Figure 2:**
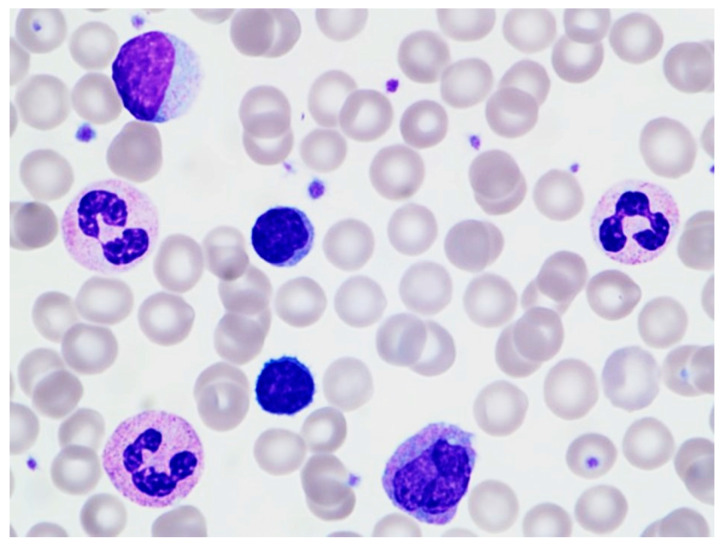
Peripheral blood morphology of transient stress lymphocytosis within 30 min of patient arrival. The smear shows small monomorphic mature-appearing lymphocytes, a large granular lymphocyte, a monocyte, and neutrophils (Wright–Giemsa stain, ×1000).

An initial complete blood count with differential (CBC/D), collected in the emergency department within 30 min of arrival, was notable for an elevated white blood cell (WBC) count of 32.3 × 10^9^/L, with an absolute neutrophil count (ANC) of 18.8 × 10^9^/L, absolute lymphocyte count (ALC) of 10.0 × 10^9^/L, monocyte count of 2.90 × 10^9^/L, eosinophil count of 0.50 × 10^9^/L, and basophil count of 0.2 × 10^9^/L. (The reference range cutoffs for lymphocytosis at our institution are ALC of 6.5 × 10^9^/L and 3.3 × 10^9^/L for 6–11-year-old pediatric patients and adult patients, respectively.) A morphologic review of the peripheral blood smear revealed predominantly small lymphocytes and neutrophilia. Large granular lymphocytes (LGLs) were present, but not increased (Figure 2).

**Figure 3:**
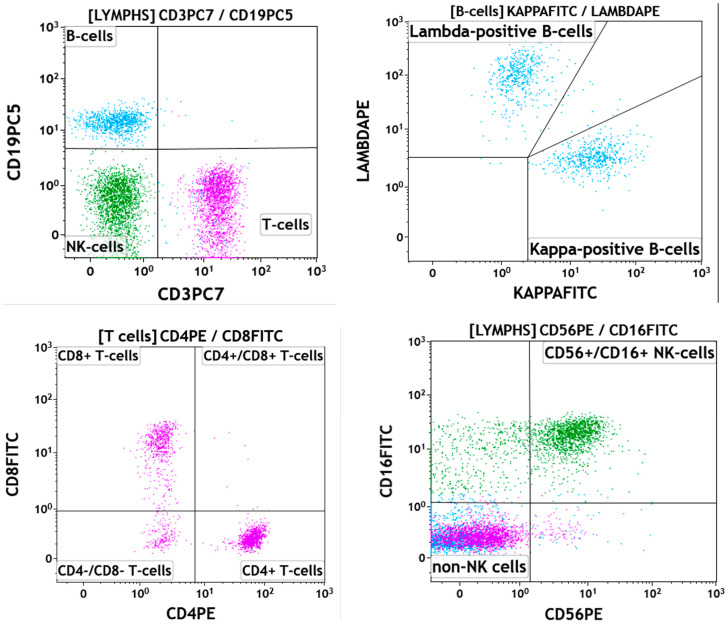
Flow cytometry plots at presentation: There is an absolute lymphocytosis, composed of a combination of B-cells (light-blue events), T-cells (pink events), and NK-cells (green events). The CD19+ B-cells are polytypic/polyclonal with a kappa-to-lambda ratio of 1.2:1. CD3+ T-cells are an admixture of CD4+ and CD8+ T-cells, with a normal CD4:CD8 ratio of 1.8:1. NK-cells are CD56+ and CD16+. There are no immunophenotypically aberrant T-cell, B-cell, or NK-cell populations.

Flow cytometric immunophenotyping studies detected 36% lymphocytes composed of 18% B-cells (CD19+, CD20+), 41% T-cells (CD3+) with a CD4:CD8 ratio of 1.8, and 27% natural killer cells (CD3−, CD56+ and/or CD57+). Neither a monoclonal B-cell population nor an immunophenotypically abnormal T-cell population was identified (Figure 3).

**Figure 4:**
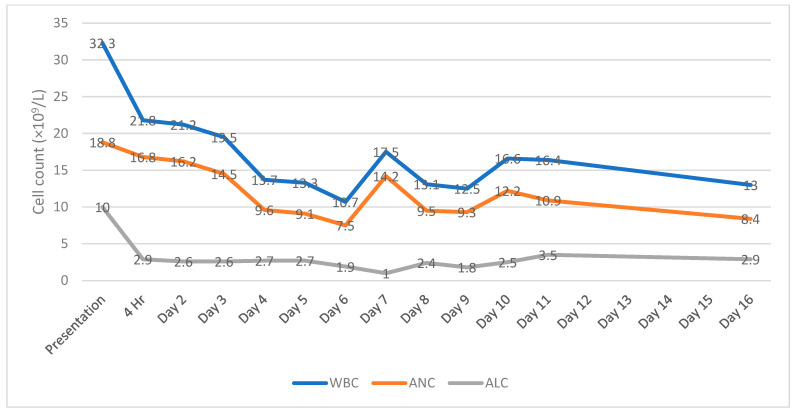
WBC, ALC, and ANC values over the course of the hospitalization. WBC, white blood cell count; ANC, absolute neutrophil count; and ALC, absolute lymphocyte count.

A CBC/D collected four hours later showed resolution of the lymphocytosis, supporting the diagnosis of TSL (Figure 4).

**Figure 5:**
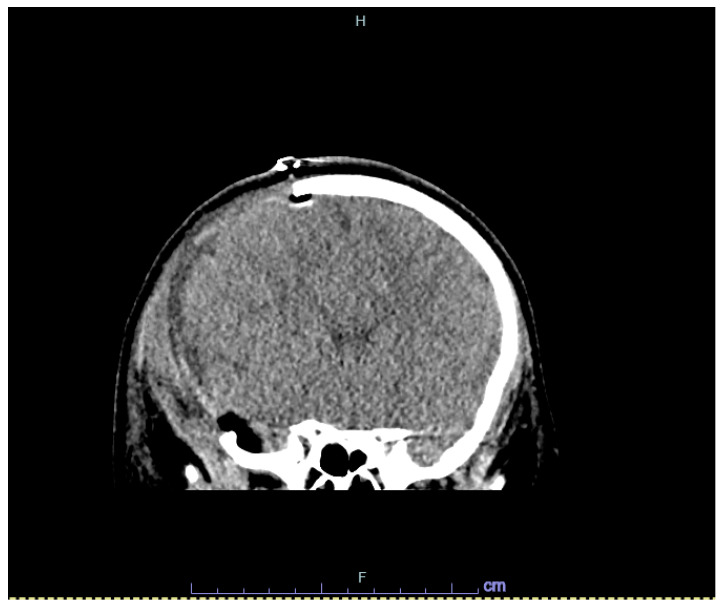
Post-hemicraniectomy head CT. Coronal view after subdural hematoma evacuation.

An emergent right hemicraniectomy with subdural hematoma evacuation was successfully performed. A subsequent head CT demonstrated improvement in the subdural hemorrhage and midline shift (Figure 5).

The WBC and ANC remained elevated for the duration of the admission, while the ALC remained within the reference range from 1.0 to 3.5 × 10^9^/L (Figure 4). Flow cytometric analysis on day 16 revealed a significant decrease in B-cells, T-cells, and NK-cells, all of which had returned to normal levels [13]. Neither a monoclonal B-cell population nor an immunophenotypically abnormal T-cell population was identified. The patient was transferred to a rehabilitation facility after two weeks with stable vital signs for continued care provided by neurosurgical and orthopedic specialists.

## 3. Systematic Review of the Literature

**Figure 6:**
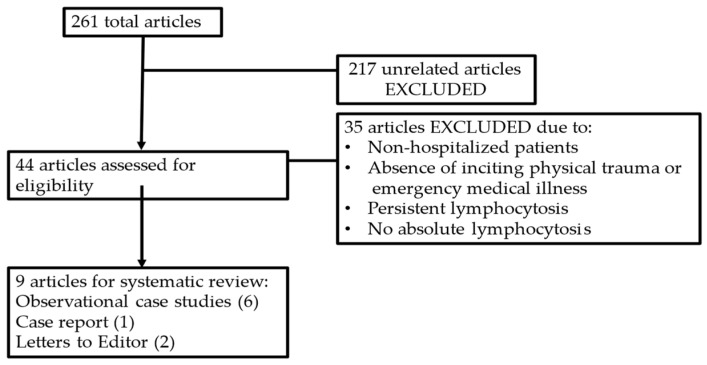
Results of systematic review of the literature.

A systematic review of the literature was performed using the PubMed database (https://pubmed.ncbi.nlm.nih.gov (accessed on 20 April 2024)). The keywords used were Stress AND Lymphocytosis, OR Transient AND Lymphocytosis. The search was filtered for human subjects only. All articles reporting cases of transient and self-resolving lymphocytosis as a result of significant physical trauma (e.g., motor vehicle accident) or emergency medical conditions (e.g., sickle cell disease crisis and myocardial infarction) were included. Articles were excluded based on the following criteria: (1) lack of absolute lymphocytosis, (2) persistent lymphocytosis, (3) healthy or non-hospitalized patients, and (4) lymphocytosis unrelated to significant physical trauma or emergency medical conditions. The titles and abstracts were reviewed to identify relevant articles and exclude others as necessary. The selected reports include observational studies, a single case report, and letters to the editor (Figure 6).

## 4. Results

We identified nine reports that met our criteria (Table 1). All articles describe TSL as a phenomenon with an elevated ALC (>5 × 10^9^/L) that spontaneously resolved within 24–48 h of injury [4,5,6,7,8,9,10,11,12]. Resolution of the lymphocytosis may occur as quickly as 75 min [6]. Despite normalization of the ALC, an elevated WBC count that remains elevated for a longer duration may persist due to a delayed rise of the ANC; this finding was reported in 32 (16%) of the 196 total cases [4,6,8]. The articles describe etiologies including significant physical injuries [7,8,11,12] and a variety of emergency medical conditions including cardiac emergencies, seizure disorders, sickle cell disease with vaso-occlusive crisis, gynecologic/obstetric emergencies, thermal injury, acute pancreatitis, hypertensive crises, rupture of cerebral artery aneurysm, and anaphylaxis [4,5,6,8,10,12].

The morphologic appearance of the lymphocytes varied between studies. Karandikar et al. and Thommasen et al. describe mature-appearing small lymphocytes on microscopic examination and increased B-cells, T-cells, and natural killer cells and a preserved CD4:CD8 ratio on flow cytometry [4,8]. Karandikar et al., Vas et al., and Groom et al. describe reactive lymphocytes (Downey type II cells), or pleomorphic lymphoma-like cells (Downey type III reactive lymphocytes) [4,5,11]. Nevertheless, even in cases where “lymphoma-like” cells were noted, none of the patients were ultimately diagnosed with lymphoma or malignancy.

We found three articles in our systematic review that document TSL in the pediatric setting [4,7,8]. Except for the study by Karandikar et. al., which mentioned the presence of reactive and lymphoma-like cells, information is limited to the severity of the physical injury, without morphologic descriptions to assist in differentiating TSL from other causes of lymphocytosis [7].

Our formal literature review excluded non-traumatic/non-emergency descriptions of transient stress lymphocytosis. For completeness, we provide the following summary of the articles that were excluded, as they did not occur in traumatic/emergent settings. TSL was described in splenic contraction secondary to dasatinib [14], acute illness during chronic lymphocytic leukemia [15], exposure to catecholamines [16,17] and glucocorticoids [18], early-phase infection of human immunodeficiency virus [19], infection with human T-lymphotropic virus type 1 [20], reactivation of Epstein Barr virus or hepatitis B virus after allogeneic hematopoietic stem cell transplantation [21,22], as an effect of anti-cancer medications such as methotrexate [23] and tirabrutinib [24], and in the recovery phase of the recently described multisystem inflammatory syndrome in children (MIS-C) associated with COVID-19 infection [25]. One case described transient lymphocytosis, but no cause was assigned (idiopathic) [26]. Lymphocytosis was also described as part of an expansion of large granular lymphocytes and natural killer-associated cells, which could be persistent or transient [27].

## 5. Discussion

TSL is an underrecognized condition associated with serious events including physical trauma and various medical emergencies that typically resolves within 24 to 48 h. Lymphocytosis ensues following peripheralization from the spleen, bone marrow, lymphatic tissue, and lungs [16].

It is postulated that the mechanism is driven by catecholamines and resolves due to release of glucocorticoids. Immunologic studies have demonstrated increased circulating lymphocytes following the release of endogenous catecholamines and steroid hormones which are induced by various physical and psychological stressors [28,29]. Additionally, lymphocytosis has been observed following infusion with epinephrine [17]. In animal models, adrenaline injection swiftly induces lymphocytosis due to mobilization from the bone marrow, spleen, and lymphatics [30]. On the other hand, glucocorticoid administration has been shown to cause a rapid decrease in ALC due to redistribution of circulating lymphocytes to the bone marrow [31].

Given the rarity of TSL in children, effective exclusion of an underlying neoplastic lymphoproliferative process is paramount when this phenomenon is encountered. Particularly, patients with cytopenias (thrombocytopenia and anemia), not explained by their acute presentation, should raise suspicion of a lymphoproliferative disorder, as should lymphadenopathy, splenomegaly, and/or constitutional symptoms such as fevers and weight loss [32]. Such symptoms may be particularly challenging to elicit when a child is admitted for major trauma with multiple distracting injuries; additionally, hematologic derangements such as anemia and thrombocytopenia are common in critically ill victims of trauma [33].

In the assessment of lymphocytosis with the peripheral smear exam, pathologists and clinicians should be aware that TSL can show a spectrum of lymphocytes that includes small lymphocytes, reactive lymphocytes, and even lymphoma-like cells [11]. Reactive lymphocytes come in several forms, which have historically been categorized into three types: Downey type I, type II, and type III. Downey type I cells are smaller cells with round to reniform or lobated nuclei with visible nucleoli, and variably basophilic to foamy to vacuolated cytoplasm. Downey type II cells are characteristic of the so-called “atypical lymphocytes” associated with infectious mononucleosis; these are large lymphocytes with loosely clumped chromatin and abundant pale cytoplasm, with radial gradation of cytoplasmic basophilia and red cells indenting their cytoplasm. Downey type III cells are “lymphoma-like” cells as they are medium to large pleomorphic cells with sometimes irregular nuclear contours, moderately coarse chromatin, and prominent nucleoli; they have moderate to abundant and deeply basophilic cytoplasm [34]. Viewing a single Downey type III cell in isolation may lead to misinterpretation of lymphocytosis as circulating lymphoma cells.

Recognizing the heterogeneity and spectrum of morphology in the lymphocytes can aid in the correct interpretation of a reactive process, as lymphoma cells are generally monomorphous [35]. Another clue to support a reactive process is the simultaneous expansion of lymphocytes and monocytes. Time permitting, serial evaluation of peripheral blood counts and smears can be particularly enlightening. On the other hand, concerning findings on initial blood counts may co-exist with falsely alarming peripheral blood smear findings, such as monomorphous lymphocytes, atypical lymphocytes, or lymphoma-like cells (even when the visual exam is performed by experienced consultants), and serial evaluation alone may not be deemed optimal; this was true for our patient, as well as for several other cases in our review of the literature [5,6,12]. In such situations where a watch-and-wait approach may not be desirable, analysis of lymphocyte subsets via flow cytometry can quickly establish the absence of a clonal lymphoproliferative disorder [4].

In our patient, the presence of a relatively monomorphous population of lymphocytes raised the possibility of lymphoproliferative disease. Easily identifiable LGLs in our patient’s peripheral blood also raised concern. In pediatric patients, clonal T-cell LGLs are associated with a wide variety of immune dysregulation states and lymphoproliferative disorders, such as common variable immunodeficiency, Burkitt lymphoma, and Hodgkin lymphoma [36]. Careful consideration with further work-up may prevent the missed opportunity of diagnosing an underlying disease, particularly in the emergency department setting in which patients may be lost to follow-up. Ultimately, we chose to pursue flow cytometry early in the patient’s course to provide additional evidence to support reactive lymphocytosis rather than lymphoma, leukemia, or associated conditions such as immune dysregulation states. The absence of clonality or an immunophenotypically abnormal population was consistent with a reactive process. Furthermore, TSL is supported by flow cytometric detection of combined (rather than isolated) expansions of B-cell, T-cell, and NK-cell populations [4,5]. These findings may be reflected morphologically by increased small lymphocytes (B- and T-cells) and LGLs (NK- and T-cells), as seen here.

Our case draws attention to TSL in childhood and includes detailed laboratory, morphologic, and flow cytometric findings. TSL is an uncommon and overlooked cause of lymphocytosis in adults. Recognition of TSL in children is important because it is a reactive process that is self-limited, does not require further work-up, and can offer reassurance to patients and their families. Although flow cytometry may be the most sophisticated technique to distinguish TSL from a lymphoproliferative disorder, the more cost-effective approach is to trend the CBC/Ds. TSL will generally resolve within 48 h. However, persistent lymphocytosis should raise suspicion for an alternate etiology that may warrant further investigation. One can avoid misdiagnosis of lymphoproliferative disease by integrating clinical history, surveillance of the CBC with differential counts, and requesting a blood smear review by an experienced hematologist, hematopathologist, and/or laboratorian.

## Figures and Tables

**Table 1:** Summary of articles documenting transient stress lymphocytosis associated with trauma or acute medical conditions.

Author (Year)	# Cases	Mean Age (Range)	Sex	ALC (× 10^9^/L) (Mean)	Etiology	Morphology	Flow Cytometric Findings	Time to Resolution
Thommasen (1986) [8]	24	(13–65)	-	>5	Physical trauma	Small lymphocytes	-	6–24 h
Teggatz (1987) [6]	26	(43–93)	-	4.1–12.9 (6.8)	Various acute medical conditions	Small lymphocytes, reactive lymphocytes, LGLs	-	75 min (earliest)
Pinkerton (1989) [7]	23	38 (16–78)	17M 6F	4.5–8.8 (5.5)	Physical trauma	-	-	
Groom (1990) [5]	25	(21–58)	14M 11F	5.1–11.1	Physical trauma, Various acute medical conditions	Reactive lymphocytes, LGLs, lymphoma-like	↑ B and T-cells ↓ CD4:CD8	10 to 24 h
Kho (1990) [9]	17	38.8	-	4–11.5 (6.9)	Surgical removal of thyroid nodules	-	-	48 h
Vas (1990) [11]	17		-	5.0–13.4 (8.5)	Various acute medical conditions	Normal, lymphoma-like, or reactive lymphocytes	↑ B-cell ↑ CD4:CD8	24 h
Wentworth (1991) [12]	8	(27–66)	-	4.2–8.5	Physical trauma, Various acute medical conditions	-	-	Majority 24 h
Siddiqui (1997) [10]	1	78	F	5.4	Middle cerebral artery aneurysmal rupture	Reactive lymphocytes	-	36 h
Karandikar (2002) [4]	52	41.4 (15–89)	22M 30F	4–10.4 (5.1)	Physical trauma, Various acute medical conditions	Small/medium sized lymphocytes +/− Reactive/LGLs/lymphoma-like	↑ B, T and NK-cells ↑ CD4:CD8	32 h Average

Acute medical conditions included cardiac emergencies (acute myocardial infarctions, cardiac arrest), gynecologic/obstetric emergencies, psychiatric emergencies, thermal trauma, seizure disorders, sickle cell disease with vaso-occlusive crisis, acute drug toxicity, acute infection, viral syndrome, cirrhosis, acute pancreatitis, anaphylaxis, hypertensive crisis, brain stem infarction, and gastroesophageal reflux disease. ALC, absolute lymphocyte count; CD, cluster of differentiation; F, female; h, hours; LGL, large granular lymphocyte; M, male; NK, natural killer; ↑, increased; ↓, decreased.

## Data Availability

Dataset available on request from the authors.
